# Utilization pattern of traditional Chinese medicine for liver cancer patients in Taiwan

**DOI:** 10.1186/1472-6882-12-146

**Published:** 2012-09-05

**Authors:** Yueh-Hsiang Liao, Cheng-Chieh Lin, Tsai-Chung Li, Jaung-Geng Lin

**Affiliations:** 1 Graduate Institute of Chinese Medicine, College of Chinese Medicine, China Medical University, Taichung, Taiwan; 2 Department of Family Medicine, China Medical University Hospital, Taichung, Taiwan; 3 School of Medicine, College of Medicine, China Medical University, Taichung, Taiwan; 4 Graduate Institute of Biostatistics, College of Public Health, China Medical University, Taichung, Taiwan; 5 Department of Health Care Administration, College of Health Science, Asia University, Taichung, Taiwan

## Abstract

**Background:**

Traditional Chinese Medicine (TCM) is one of the most popular complementary and alternative medicine modalities worldwide. In Chinese and East Asian societies, TCM plays an active role in the modern health care system and is even covered by the National Health Insurance Program of Taiwan. Liver cancer is the second most common cancer in Taiwan. This study aimed to analyze the TCM utilization patterns of patients with liver cancer from 1996–2007 using a population-based random sample of one million insured patients.

**Methods:**

A cross-sectional study was conducted using registration and claim data sets from 1996 to 2007 obtained from the Longitudinal Health Insurance Database 2005 (LHID2005). The outpatient datasets contained the encounter form-based dates of visit, three items from the International Classification of Diseases (Ninth Revision, Clinical Modification codes), the primary procedure (e.g., drug or diagnostic procedure), type of copayment, billed amount, and paid amount. Only ambulatory care was analyzed.

**Results:**

A total of 6358 liver cancer patients utilized ambulatory care during the study period. Among them, 1240 (19.50%) availed of TCM outpatient services. The prevalence of TCM use fluctuated during the study period, with a peak of 25.11% in 2001. After multivariable adjustment, the likelihood of TCM users was lower in participants aged 70 years and older (odds ratio, OR = 0.79, 95% confidence interval, CI: 0.64–0.97), males (OR = 0.60, 95% CI: 0.52–0.68), residents of Taipei (OR = 0.75, 95% CI: 0.58–0.96) as well as farmers and fishermen (OR = 0.71, 95% CI: 0.54–0.94), but was higher in residents of central Taiwan (OR = 1.99, 95% CI: 1.56–2.54. Most biomedicine and TCM outpatient services were provided by private clinics, followed by private hospitals. The two most frequently recorded coexisting diseases for both biomedicine and TCM outpatient visits specifically for liver cancer were (1) chronic liver disease and cirrhosis, and (2) malignant neoplasm of the liver and hepatic bile duct. The mean fee per visit for biomedicine was much higher than that for TCM, and the average expenditure was NT$429.73 (US$13.25) per biomedicine visit and NT$301.93 (US$9.32) per TCM visit (US$1 = NT$32.4 in 2007). For outpatient visits specifically for liver cancer, the mean fee per visit for biomedicine was much higher than that for TCM. The average cost per visit was NT$1457.31 (US$44.98) for biomedicine and NT$1080.76 (US$33.36) for TCM.

**Conclusion:**

TCM was widely used by the patients with liver cancer, and the prevalence of TCM use remained stably high during the study period. The costs of insurance covering TCM were consistently lower than those covering biomedicine in patients with liver cancer. The findings of this study should be useful for health policy makers as well as researchers considering the integration of TCM and biomedicine.

## Introduction

### Incidence and mortality of liver cancer (LC)

LC is the leading cause of cancer-related mortality worldwide. Mongolia, Gambia, Taiwan, China, and Thailand have the highest incidence rates of LC in the world, and their age standardized incidence rates in 2008 were 94.4, 36.1, 35.7, 33.8, and 29.3 per 100 000 persons, respectively [1]. In Taiwan, LC accounts for more than 28% of total deaths [2], and ranks as the second most common cancer in both men and women, accounting for about 20% of all cancer deaths. The annual standardized mortality incidence increased from 22.7/100 000 in 1991 to 26.2/100 000 in 2009 [2]. In China, LC accounts for more than 10% of total deaths and ranks as the fourth most common cancer [3].

### Use of Complementary and Alternative Medicine worldwide

The use of Complementary and Alternative Medicine (CAM) has gained worldwide popularity. The motives for the use of CAM include perceived failure of standard health care, the need of a patient for autonomy, and preference for holistic or natural therapy in Western populations [4-6]. CAM is commonly used together with conventional medicine and has entered mainstream society and culture [7-9]. According to the 2007 National Health Interview Survey, the prevalence of CAM use is about 38% in American adults [9]. In a survey across a number of European countries, the percentages of CAM use range from 22.7% for head and neck cancer patients to 56.3% for pancreatic cancer patients [10].

### Use of Traditional Chinese Medicine (TCM) in Asian countries

TCM is one of the most popular CAM forms worldwide. The motives for TCM use include cultural belief about TCM in managing illness symptoms, maximizing conventional treatment effect, and preventing recurrence in Chinese populations [11-13]. TCM is commonly used together with conventional medicine and has entered mainstream society and culture. In Chinese and East Asian societies, TCM plays an active role in the modern health care system. Unlike CAM that is not funded by either private or social insurance companies in most Western societies, TCM is covered by the National Health Insurance (NHI) Program of Taiwan. Thus, one important feature of the NHI Program is the coverage of both biomedicine (Western) and TCM. By 2003, after implementing the NHI Program, more than 99% of the 23 million persons residing in Taiwan had been covered by this universal health insurance plan. Compared with CAM use by Western populations, TCM has a higher level of accessibility due to lesser financial barrier. A study on the determinants of TCM and acupuncture utilization of cancer patients in Taiwan has shown that the prevalence of TCM ranges from 14.81% for cervical cancer to 30.13% for breast cancer [8].

### Therapeutic effect of TCM against hepatocellular carcinoma (HCC) in previous studies

Treatment with TCM has been investigated for its effect on the stimulation of host immune response that has cytotoxic activity against HCC. TCM is commonly used in combination with transcatheter arterial chemoembolization (TACE) to treat patients with unresectable HCC [14-16]. The findings of a meta-analysis of 37 randomized controlled trials involving 2653 patients reveal that TCM plus TACE improves patient survival, quality of life, alleviation of symptoms, and tumor response [14]. In another meta-analysis, TCM plus TACE significantly increases the survival, complete or partial response, non-deterioration performance, T cells, natural killer cells, and white blood cell count; significantly decreases the level of blood alpha-fetoprotein concentration; and significantly lowers the risk for nausea and vomiting [15]. A recent meta-analysis has also demonstrated that TCM plus TACE increases the proportions of cluster differentiation (CD) 3(+) T cells, CD4(+) T cells, and natural killer cells, as well as the ratio of CD4(+)/CD8(+) before and after treatment [16]. A meta-analysis of randomized controlled trials for TCM combined with chemotherapy has reported promising evidence that TCM plus chemotherapy may benefit patients with HCC [17]. The most commonly used ingredients demonstrated to have oncologic and immunologic pharmacology are *AstragalusPanax ginseng*, toad skin secretions (bufotoxin), beetle extracts (*Mylabris*), *Atractylodes, Bupleurum*, and *Curcuma*[18].

### How the current study helps resolve the uncertainties regarding TCM use

Previous studies have explored the utilization of TCM either in a general population [19-24], in a single clinical setting [25], or for acupuncture use only [26]. Two studies have described the use frequencies, characteristics of users, and disease categories treated by TCM in Taiwan from 1996 to 2001 [27] and from 1997 to 2003 [28] using complete NHI datasets for TCM. A recent study has explored TCM use among all prostate cancer patients in the NHI datasets in 2007 [29]. However, TCM use among patients with LC has not yet been reported. This study aimed to compare the differences between the characteristics, types of care provider, existing diseases, and expenditures for outpatient services of TCM and non-TCM users with LC enrolled in the NHI Program in Taiwan from 1996 to 2007 using a population-based random sample of one million insured patients.

## Methods

### Data sources

The NHI program was initiated in Taiwan in 1995 and currently covers about 99% of the 23 million population of Taiwan. The national government-run Bureau of National Health Insurance (BNHI) has contracted with 97% of all hospitals and 92% of all clinics nationwide. The BNHI conducts an expert review of random samples of every 50–100 ambulatory and inpatient claims in each hospital and clinic quarterly, and false reports of diagnosis are severely penalized [30]. The NHI Research Database (NHIRD) provides registration and claim datasets from a random sample of one million beneficiaries for use in research. The data for the present study was obtained from the Longitudinal Health Insurance Database 2005 (LHID2005), which was released by the Taiwan National Health Research Institute in 2007. The LHID2005 contains all ambulatory and inpatient claim data of one million beneficiaries who were randomly sampled from 23 million enrollees in the NHI. The distributions of the beneficiary age and gender in the LHID2005 and original NHI database are similar.

A retrospective study was conducted using the registration and claim datasets for the years 1996–2007 from the LHID2005. The LHID2005 database contains comprehensive information, such as demographic data, dates of clinical visits, diagnostic codes, expenditure amounts, and others. The amount of expenditure in the dataset was that covered by the NHI Program, which represented the consumption of medical resources. The NHI Program has a committee that reviews new treatments, drugs, and procedures, and only those that prove to have evidence-based effectiveness are covered. The expenditure can be classified into the following categories: fees for consultation, treatment, and medical supply; diagnosis fee; and drug fee. Files of registry for beneficiaries, ambulatory care expenditures by visits, and inpatient care by admission were obtained for analysis. To protect privacy, data on the patient identities and institutions were scrambled cryptographically by the NHIRD. Patients’ privacy is under protection form using these data. Our study using these data are exempted from institutional review board approval of Public Health, Social and Behavioral Science Committee Research Ethics Committee, China Medical University and Hospital.

The NHI covered TCM outpatient care, but not inpatient care. Therefore, only TCM and biomedicine ambulatory care were analyzed in this study. The outpatient datasets contained encounter form-based date of visits, patient gender and date of birth, medical facility visited, department visited, prescribing physician, dispensing pharmacist, three items from the International Classification of Diseases, Ninth Revision, Clinical Modification (ICD-9-CM) codes, primary procedure (e.g., drug or diagnostic procedure), type of copayment, billed amount, and paid amount.

### Statistical analysis

Continuous variables were reported as mean and 95% confidence interval (CI), and categorical variables were reported as number, percentage, and 95% CI. Differences in proportions and means were assessed using a χ^2^-test or a *t-*test. Adjusted odds ratios (ORs) were estimated by multivariate logistic regression analysis. Simple linear regression was used to examine the annual trend of TCM use in 1996–2007. All reported p values were those of two-sided tests. The statistical significance was set at p < 0.05. All analyses were performed using SAS version 9.1 (SAS Institute Inc., Cary, NC, USA).

## Results

### Prevalence of TCM use over time

**Figure 1:**
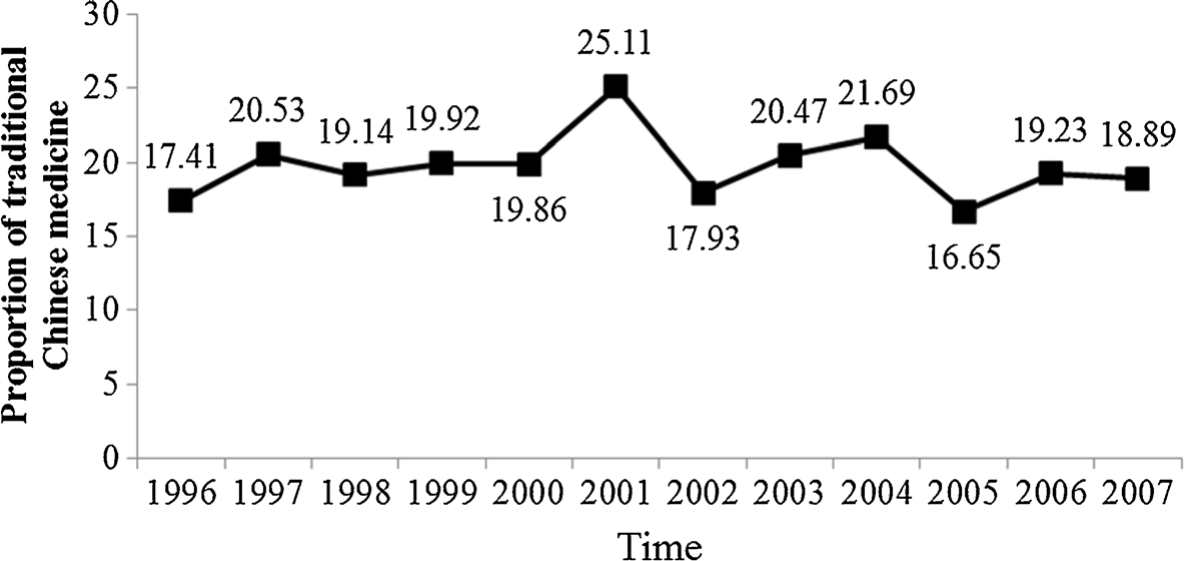
Proportion of traditional Chinese medicine use by liver cancer patients during the years 1996–2007.

A total of 6,358 LC patients used ambulatory care in 1996–2007. Among them, 1,240 (19.50%) used TCM outpatient services. Figure 1 shows the prevalence of TCM use during the study period. The prevalence of TCM use fluctuated and had a peak of 25.11% in 2001. There was no significant linear trend (R^2^ = 0.0029, β = −0.0369, p = 0.8689).

### Factors associated with TCM use

The demographics are presented in Table 1 according to the status of TCM use. The mean patient age was 57.95 years (SD = 11.46) for TCM users and 59.65 years (SD = 12.30) for non-users. After multivariable adjustment, the likelihood of TCM users was lower in participants aged 70 years and older (odds ratio, OR = 0.79, 95% confidence interval, CI: 0.64–0.97, males (OR = 0.60, 95% CI: 0.52–0.68), residents of Taipei (OR = 0.75, 95% CI: 0.58–0.96) as well as farmers and fishermen (OR = 0.71, 95% CI: 0.54–0.94), but was higher in residents of central Taiwan (OR = 1.99, 95% CI: 1.56–2.54). There was no significant difference between the insured amount and urbanization level of TCM users and nonusers. We further categorized TCM users into TCM only users and TCM plus biomedicine users. Their distributions along with those of biomedicine only users are shown in Additional file 1.

**Table 1:** Liver cancer patient characteristics during the period 1996–2007

**Characteristic**	**TCM Nonusers**		**TCM Users**		**Adjusted**^**a**^**OR**	**95% CI**
	**Total**	**%**	**Total**	**%**		
Patient no.	5118		1240			
Age	59.65 ± 12.30		57.95 ± 11.46			
<50 (reference)	1315	25.69	343	27.66	1.00	
50s	1283	25.07	383	30.89	1.15	0.97-1.37
60s	1274	24.89	275	22.18	0.84	0.69-1.02
> = 70s	1246	24.35	239	19.27	0.79	0.64-0.97
Gender						
Female (reference)	1957	38.24	631	50.89	1.00	
Male	3161	61.76	609	49.11	0.60	0.52-0.68
Insured amount (NT$/month)						
<20,000 (reference)	3491	68.21	815	65.73	1.00	
20,000-39,999	964	18.84	245	19.76	0.92	0.77-1.11
40,000-59,999	459	8.97	121	9.76	1.01	0.78-1.30
> = 60,000	204	3.99	59	4.76	1.21	0.87-1.70
Urban Level						
1 (reference)	1456	29.19	348	28.74	1.00	
2	1461	29.29	358	29.56	0.91	0.75-1.10
3	708	14.19	187	15.44	0.91	0.71-1.15
4	817	16.38	201	16.6	0.85	0.66-1.08
> = 5	546	10.95	117	9.67	0.80	0.58-1.08
Residential Area						
Northern (reference)	667	13.03	149	12.02	1.00	
Taipei	1923	37.57	383	30.89	0.75	0.58-0.96
Central	709	13.85	306	24.68	1.99	1.56-2.54
Southern	931	18.19	215	17.34	1.12	0.86-1.44
Eastern	89	1.74	28	2.26	1.40	0.83-2.36
Kao-Ping	783	15.3	153	12.34	0.91	0.69-1.18
Insured unit						
Government, school employees (reference)	530	10.93	158	13.4	1.00	
Private enterprise employees	1390	28.68	361	30.62	0.92	0.73-1.15
Member of occupational	960	19.81	271	22.99	0.99	0.77-1.27
Farmers, fishermen	1215	25.07	246	20.87	0.71	0.54-0.94
Low-income households Veterans, other regional	752	15.51	143	12.13	0.80	0.60-1.07

TCM: traditional Chinese Medicine; OR: odds ratio; CI: confidence interval.

a: Adjusted ORs were from the model considering age, gender, insured amount, residential area, and insured unit.

### Medical institutes

Most biomedicine outpatient services were provided by private clinics (39.71%), followed by private hospitals (39.4%) (Table 2). Similarly, the majority of TCM outpatient services were provided by private clinics (52.69%) and private hospitals (30.99%). The remaining TCM outpatient services were provided by public hospitals (8.98%). The proportion of TCM outpatient services provided by private clinics was much higher than that of biomedicine outpatient services provided by private clinics (52.69% vs. 39.71%, p < 0.001).

**Table 2:** Liver cancer outpatient service providers during the period 1996-2007

**Outpatient visits specifically for liver cancer Characteristic**	**Biomedicine**		**TCM users**		**P value for**
	**Visits**	**Percentage (95%CI)**	**Visits**	**Percentage (95%CI)**	**χ**^**2**^
Type of providers					<0.001
Public hospitals	75716	11.91 (11.83-11.99)	20455	8.98 (8.86-9.1)	
Public Chinese medicine hospitals	34	0.01 (0.01-0.01)	636	0.28 (0.26-0.30)	
Private hospitals	250469	39.40 (39.28-39.52)	70631	30.99 (30.80-31.18)	
Private Chinese medicine hospitals	326	0.05 (0.04-0.06)	2754	1.21 (1.17-1.25)	
Public clinics	20175	3.17 (3.13-3.21)	4253	1.87 (1.81-1.93)	
Private clinics	252442	39.71 (39.59-39.83)	120067	52.69 (52.49-52.89)	
Unknown	36543	5.75 (5.69-5.81)	9098	3.99 (3.91-4.07)	
Total	635705	100	227894	100	

TCM: traditional Chinese medicine; CI: confidence interval.

### Existing diseases

The diagnoses in all ambulatory claim data were recorded in the ICD-9-CM format. The five most frequently recorded existing disease codes for biomedicine and TCM outpatient visits specifically for LC as well as for all outpatient visits are presented in Table 3. For outpatient visits specifically for LC, the four (out of five) most frequently recorded existing diseases for both biomedicine and TCM were (1) chronic liver disease and cirrhosis, (2) malignant neoplasm of the liver and hepatic bile ducts, (3) essential hypertension, and (4) acute upper respiratory infections. In contrast, diabetes mellitus was the third most frequently recorded existing disease for biomedicine, whereas general symptoms were the third most frequently recorded existing disease for TCM. For all outpatient visits, the four (out of five) most frequently recorded existing diseases for both biomedicine and TCM were (1) acute upper respiratory infection, (2) essential hypertension, (3) diabetes mellitus, and (4) general symptoms. In contrast, conjunctival disorders was the fifth most frequently recorded existing disease for biomedicine, whereas other and unspecified disorders of the back were the fourth most frequently recorded existing disease for TCM.

**Table 3:** Top 5 disease codes among liver cancer patients during the years 1996–2007 for all outpatient visits and outpatient visits specifically for liver cancer

	**Western medicine**			**Traditional chinese medicine**		
**Ranking**	**Disease (Code)**	**No.**	**Percentage (95% CI)**	**Disease (Code)**	**No.**	**Percentage (95% CI)**
**Outpatient visits specifically for liver cancer**						
**Total = 1,181,130**				**Total = 384,074**		
1	Chronic liver disease and cirrhosis (571)	84,263	7.31 (7.26-7.36)	Chronic liver disease and cirrhosis (571)	22,419	5.84 (5.77-5.91)
2	Malignant neoplasm of the liver and hepatic bile ducts (155)	75,881	6.42 (6.38-6.46)	Malignant neoplasm of the liver and hepatic bile ducts (155)	1,9847	5.17 (5.10-5.24)
3	Diabetes Mellitus (250)	49,506	4.19 (4.15-4.23)	General symptoms (780)	13,060	3.4 (3.34-3.46)
4	Essential hypertension (401)	48,762	4.13 (4.09-4.17)	Acute upper respiratory infections (465)	12,231	3.18 (3.12-3.24)
5	Acute upper respiratory infections (465)	40,502	3.43 (3.40-3.46)	Essential hypertension (401)	11,604	3.02 (2.97-3.07)
**Ranking**	**Disease (Code)**	**No.**	**Percentage (95%CI)**	**Disease (Code)**	**No.**	**Percentage (95%CI)**
**All outpatient visits**						
**Total = 86,850,726**				**Total = 29,277,941**		
1	Acute upper respiratory infections (465)	5,932,918	6.84 (6.83-6.85)	Acute upper respiratory infections (465)	1,757,625	6.00 (5.99-6.01)
2	Essential hypertension (401)	3,884,150	4.47 (4.47-4.47)	General symptoms (780)	857,752	2.93 (2.92-2.94)
3	Diabetes Mellitus (250)	3,027,309	3.49 (3.49-3.49)	Essential hypertension (401)	805,130	2.75 (2.74-2.76)
4	General symptoms (780)	2,030,401	2.34 (2.34-2.34)	Other and unspecified disorders of the back (724)	706,248	2.41 (2.40-2.42)
5	Disorders of conjunctica (372)	1,642,787	1.89 (1.89-1.89)	Diabetes Mellitus (250)	635,054	2.17 (2.16-2.18)

CI: confidence interval.

### Expenditures

Table 4 shows the details of expenditures. Biomedicine outpatient services accounted for 72.73% of all outpatient visits and 77.61% of total expenditures, whereas TCM outpatient services accounted for 27.28% of visits and 22.39% of expenditures. The fees for consultation, treatment, and medical supplies, as well as drugs per visit for biomedicine were markedly higher than those for TCM. The average expenditure was NT$429.73 (US$13.25) per biomedicine visit and NT$301.93 (US$9.32) per TCM visit, and the average cost per visit was NT$981.01 (US$30.28) for biomedicine and NT$754.38 (US$23.28) for TCM (p < 0.001 for all comparisons) (US$1 = NT$32.4 in 2007). The ratio of medical expenses for all biomedicine outpatient visits relative to that for TCM was 3.47, whereas the ratio of the average medical expenses for each biomedicine outpatient visit relative to that for TCM was 1.3.

**Table 4:** Expenditures for outpatient services for liver cancer patients (NT$) during the period 1996-2007

	**Biomedicine (N = 53999777)**			**TCM (N = 20253460)**				**Biomedicine/ TCM Ratio**	
**Characteristic**	**Total**	**Percentage (95% CI)**	**Average (95% CI)**	**Total**	**Percentage (95% CI)**	**Average (95% CI)**	**t value**	**Total**	**Average**
Medical expenses for all outpatient visits									
Fees for consultation, treatment, andmedical supply	23,205,324,170	43.8 (43.8-43.8)	429.73 (429.71-429.75)	6,115,161,733.80	40.02 (40.02-40.02)	301.93 (301.90-301.96)	226.64*	3.79	1.42
Diagnosis fee	11,725,511,578	22.13 (22.13-22.13)	217.14 (217.14-217.14)	4,425,149,296	28.96 (28.96-28.96)	218.49 (218.49-218.49)	−59.07*	2.65	0.99
Drug fee	18,043,485,487	34.06 (34.06-34.06)	334.14 (334.13-334.15)	4,738,494,125	31.01 (31.01-31.01)	233.96 (233.95-233.97)	388.04*	3.81	1.43
Total amount	52,974,321,235	100	981.01 (980.98-981.04)	15,278,805,154.80	100	754.38 (754.35-754.41)	365.10*	3.47	1.30
	**Biomedicine (N = 635705)**			**TCM (N = 227894)**				**Biomedicine/ TCM Ratio**	
**Characteristic**	**Total**	**Percentage (95% CI**)	**Average (95% CI)**	**Total**	**Percentage (95% CI)**	**Average (95% CI)**	**t value**	**Total**	**Average**
**Medical expenses for outpatient visits specifically for liver cancer**									
Fees for consultation,treatment, andmedical supply	470,152,992	50.75 (50.75-50.75)	739.58 (739.32-739.84)	115449,750	46.87 (46.86-46.87)	506.59 (506.22-506.96)	29.19*	4.07	1.46
Diagnosis fee	137,611,256	14.85 (14.85-14.85)	216.47 (216.46-216.48)	50,252,360	20.40 (20.39-20.40)	220.51 (220.5-220.52)	−16.35*	2.74	0.98
Drug fee	318,652,124	34.40 (34.40-34.40)	501.26 (501.13-501.39)	80,595,549	32.72 (32.71-32.72)	353.65 (353.44-353.86)	33.91*	3.95	1.42
Total amount	926,416,372	100	1457.31 (1457.02-1457.60)	246,297,659	100	1080.76 (1080.33-1081.19)	40.99*	3.76	1.35

TCM: traditional Chinese medicine; *:p < 0.001; CI: confidence interval.

For outpatient visits specifically for LC, biomedicine outpatient services accounted for 73.61% of all visits and 79.00% of total expenditures, whereas TCM outpatient services accounted for 26.39% of visits and 21.00% of expenditures. The fees for consultation, treatment, and medical supplies, as well as drugs per visit for biomedicine were markedly higher than those for TCM. The average expenditure was NT$739.58 (US$22.83) per biomedicine visit and NT$506.59 (US$15.64) per TCM visit. The average cost per visit was NT$1457.31 (US$44.98) for biomedicine and NT$1080.76 (US$33.36) for TCM (p < 0.001 for all comparisons). The ratio of medical expenses for all biomedicine outpatient visits relative to that for TCM was 3.76, whereas the ratio of the average medical expenses for each biomedicine outpatient visit relative to that for TCM was 1.35.

## Discussion

This study is the first large-scale survey focusing on TCM use among LC patients in Taiwan. The overall prevalence of insurance-covered TCM use in outpatient services among LC patients was 19.50% and remained stably high during the study period. TCM outpatient services accounted for 27.28% of the visits and 22.39% of the outpatient service expenditures of patients with LC. The costs of insurance covering TCM were consistently lower than those covering biomedicine.

The findings on the prevalence of TCM use in patients with LC were similar to those of previously studied patients with mild diseases or prostate cancer [28,29]. The prevalence of TCM utilization in our study was higher than that reported by [Lin et al. 29] for patients with prostate cancer (19.50% vs. 2.6%). One possible explanation is that TCM is more frequently used by patients with LC because the disease severity of LC requires more intensive treatment and patients with LC are inclined to seek alternative treatments. Another possible explanation is that only outpatient visits specific for prostate cancer have been considered in the study of [Lin et al. 29], whereas all outpatient visits were considered in the current work.

Our results suggested that TCM services were utilized more often by females and residents of central Taiwan, but less often by patients >70 years old, residents of Taipei, as well as farmers and fishermen. The higher TCM use of central Taiwan residents may probably be due to the higher availability of TCM providers in this area. Before 1998, the only medical university that provided formal TCM education was located in central Taiwan; thus, the ratio of TCM physicians to 10 000 residents was 1090 for Taichung city in central Taiwan compared with 771 for the second highest area in 2010. There are two possible reasons that can explain the low TCM use of farmers and fishermen. One is that farmers and fishermen live in areas with less access to TCM providers. Another is that their economic status is usually lower and the extra expenditure for TCM use may be a financial burden. Further research on the barriers for TCM use is warranted.

Based on the ICD-9-CM codes, we found that chronic liver disease and cirrhosis, as well as general symptoms were the primary indications for TCM. In biomedicine, the top two primary indications, apart from malignant neoplasm of the liver and hepatic bile ducts, were chronic liver disease and cirrhosis, as well as diabetes. These findings on the disease pattern of health care use may be explained by the fact that patients seek TCM to relieve symptoms. A recent systematic review of qualitative and quantitative studies regarding the views on traditional Chinese medicine amongst Chinese populations worldwide found that patients worldwide had a common cultural affinity to TCM and considered TCM as an effective complement to western medicine (WM) for treating chronic or serious diseases [31]. Their finding may explain why LC patients in Taiwan like to seek TCM treatments for relieving their “general symptoms”.

We observed that the proportions of hypertension and diabetes only accounted for a small portion of all ICD-9 codes made by patients with LC. Caution should be taken when interpreting these proportions. This proportion was affected by both the disease prevalence among Taiwanese LC patients and the rate of outpatient utilization for each disease by LC patients with co-existing conditions. This proportion is also related to insufficient comprehensive coding. For example, only three diseases were coded if a patient sought outpatient care for more than three diseases. Our data underestimated the proportion of existing disease among all ICD-9 codes.

Using data of liver cancer patients from a representative sample of Taiwan population, we found that women had higher TCM use than men. This finding is consistent with those that had been conducted in general population in Taiwan [32], patients with chronic non-communicable disease [33] and patients with schizophrenia in Hong Kong [34]. Women have been found to be more predisposed to report their health as poor, which has been hypothesized that this result from women’s health beliefs and help-seeking behavior [35]. There are two possible reasons why patients who aged 70 years and older or who were farmers and fishermen had lower TCM use than their counterparts. First, they lived in rural areas with lower density of medical services, especially TCM. Previous studies exploring the factors associated with TCM uses in Taiwan have found that people living in rural areas are less likely to access various conventional or unconventional therapies [28,36,37]. Second, the socioeconomic status (SES) of these individuals is usually low and low SES has been reported to be associated with low TCM use [38].

In Taiwan, the national health insurance program includes both biomedicine and TCM. Under two modalities of medicine within one health care system, it enhances the high availability and affordability of these two types of health care services, thus improves the quality, comprehensiveness, and efficiency of care. However, the majority of the TCM services were provided by the private sector. Public hospitals or clinics did not set up TCM departments until recent years because of popularity of TCM worldwide. Up to date, not all public hospitals or clinics provide TCM services. Most of patients who seek for health care service in public hospitals or clinics are veterans or individuals living in rural area or with low SES. It would increase the difficulty to bring biomedicine and TCM care together for those patients seeking care in public hospitals or clinics. Developing strategies to overcome the barrier for these individuals would be an important task for health care planners and policy makers.

The strength of this study is that it is the first large-scale study of TCM use in Taiwanese society, and the NHI databases are representative of the general population in Taiwan because the NHI Program covers more than 99% of Taiwan’s 23 million population and 93% of the medical institutes. Previous studies on health care utilization with regard to TCM/CAM use have been conducted with clinic attendees, telephone interviews, self-administered interviews, household interviews, as well as hospital and private clinic surveys. These studies usually have limited sample sizes, and have been conducted in countries in which TCM/CAM is not covered by insurance. Thus, the pattern and characteristics of TCM/CAM use may be affected by the socioeconomic status of individuals.

There are several limitations to this study. First, our study is a kind of administrative database research and uses ICD-9-CM codes to identify patients with LC and use drug and diagnosis procedure for care costs [39]. Misclassification errors from this type of research are inevitable. Second, some herbal medicines not covered by NHI and visits at clinics not under contract with the BNHI were not considered. About 10% of TCM clinics are not under contract with the BNHI because the NHI payment is low. Some patients may also be taking decoction forms of Chinese medicine, which is not covered by the NHI Program. Hence, the NHIRD data may lead to an underestimation of TCM costs. Third, clinical and biochemical measurements, such as staging and alpha fetal protein, were not included in the study; thus, the TCM use and costs stratified by these factors cannot be explored.

## Conclusion

The NHI Program of Taiwan is a comprehensive and universal health insurance program that covers both conventional biomedicine and TCM services. The prevalence of TCM use among LC patients remained stably high. The costs of insurance-covered TCM were consistently lower than those of biomedicine. This study provided information on TCM use frequency as well as coexisting diseases treated by biomedicine and TCM in LC patients, which should be useful to health policy makers and practitioners who consider the integration of TCM and biomedicine.

## Abbreviations

TCM: Traditional Chinese medicine; CAM: Complementary and alternative medicine; LHID2005: Longitudinal health insurance database 2005; ICD-9-CM: International classification of diseases ninth revision: clinical modification; LC: Liver cancer; NHI: National health insurance; HCC: Hepatocellular carcinoma; TACE: Transcatheter arterial chemoembolization; CD: Cluster differentiation; BNHI: Bureau of national health insurance; NHIRD: NHI research database; SD: Standard deviation.

## Competing interests

All authors have declared that they have no competing interest.

## Authors’ contributions

YHL, CCL and TCL contributed equally to the design of the study and direction of its implementation, including supervision of the field activities, quality assurance and control. YHL and JGL supervised the field activities. JGL and TCL helped conduct the literature review and prepare the Methods and Discussion sections of the text. TCL and CCL designed the study’s analytic strategy and conducted the data analysis. All authors read and approved the final manuscript.

## Pre-publication history

The pre-publication history for this paper can be accessed here:

http://www.biomedcentral.com/1472-6882/12/146/prepub

## Supplementary Material

Additional file 1: Table 1 Liver cancer patient Biomedicine and TCM services during the period 1996–2007. Click here for file
