# Supplementary material for: Utilization pattern of traditional Chinese medicine for liver cancer patients in Taiwan
Source: BMC Complement Altern Med. 2012 Sep 5;12:146. doi: 10.1186/1472-6882-12-146 (PMC3575295; doi:10.1186/1472-6882-12-146)
Supplement: Additional file 1: Table 1 — Liver cancer patient Biomedicine and TCM services during the period 1996–2007. [file 1472-6882-12-146-S1.doc]

**Additional file 1 –** Table1. Liver cancer patient Biomedicine and TCM services during the period 1996-2007

| Characteristic | Biomedicine | | TCM | | Both | | χ^2^ |
| --- | --- | --- | --- | --- | --- | --- | --- |
|  | Total | % | Total | % | Total | % |  |
| Patient no. | 5118 |  | 102 |  | 1138 |  |  |
| Age | 59.65±12.30 | | 55.03±11.32 | | 58.22±11.44 | |  |
| <50 | 1315 | 25.69 | 38 | 37.25 | 305 | 26.80 | 40.08* |
| 50s | 1283 | 25.07 | 37 | 36.27 | 346 | 20.77 |  |
| 60s | 1274 | 24.89 | 11 | 10.78 | 264 | 17.04 |  |
| >=70s | 1246 | 24.35 | 16 | 15.69 | 223 | 15.02 |  |
| Gender |  |  |  |  |  |  |  |
| Female | 1957 | 38.24 | 42 | 41.18 | 589 | 51.76 | 70.52* |
| Male | 3161 | 61.76 | 60 | 58.82 | 549 | 48.24 |  |
| Insured amount (NT$/month) |  |  |  |  |  |  |  |
| <20,000 | 3491 | 68.21 | 63 | 61.76 | 752 | 66.08 | 6.86 |
| 20,000-39,999 | 964 | 18.84 | 19 | 18.63 | 226 | 19.86 |  |
| 40,000-59,999 | 459 | 8.97 | 12 | 11.76 | 109 | 9.58 |  |
| >=60,000 | 204 | 3.99 | 8 | 7.84 | 51 | 4.48 |  |
| Urban Level |  |  |  |  |  |  |  |
| 1 | 1456 | 29.19 | 31 | 31.00 | 317 | 28.53 | 4.63 |
| 2 | 1461 | 29.29 | 33 | 33.00 | 325 | 29.25 |  |
| 3 | 708 | 14.19 | 13 | 13.00 | 174 | 15.66 |  |
| 4 | 817 | 16.38 | 13 | 13.00 | 188 | 16.92 |  |
| >=5 | 546 | 10.95 | 10 | 10.00 | 107 | 9.63 |  |
| Insured unit |  |  |  |  |  |  |  |
| Northern | 667 | 13.03 | 10 | 9.8 | 139 | 12.21 | 103.22* |
| Taipei | 1923 | 37.57 | 28 | 27.45 | 355 | 31.20 |  |
| Central | 709 | 13.85 | 20 | 19.61 | 286 | 25.13 |  |
| Southern | 931 | 18.19 | 24 | 23.53 | 191 | 16.78 |  |
| Eastern | 89 | 1.74 | 4 | 3.92 | 24 | 2.11 |  |
| Kao-Ping | 783 | 15.3 | 16 | 15.69 | 137 | 12.04 |  |
| Insured unit |  |  |  |  |  |  |  |
| Government, school employees | 530 | 10.93 | 23 | 23.47 | 135 | 12.49 | 36.27* |
| Private enterprise employees | 1390 | 28.68 | 23 | 23.47 | 338 | 31.27 |  |
| Member of occupational | 960 | 19.81 | 20 | 20.41 | 251 | 23.22 |  |
| Farmers, fishermen | 1215 | 25.07 | 20 | 20.41 | 226 | 20.91 |  |
| Low-income households Veterans, other regional | 742 | 15.51 | 12 | 12.24 | 131 | 12.12 |  |

*P<0.001
